# Microglia and Brain Disorders: The Role of Vitamin D and Its Receptor

**DOI:** 10.3390/ijms241511892

**Published:** 2023-07-25

**Authors:** Alessandra Mirarchi, Elisabetta Albi, Tommaso Beccari, Cataldo Arcuri

**Affiliations:** 1 Department of Medicine and Surgery, University of Perugia, 06123 Perugia, Italy; alessandra.mirarchi@studenti.unipg.it; 2 Department of Pharmaceutical Sciences, University of Perugia, 06123 Perugia, Italy; elisabetta.albi@unipg.it (E.A.); tommaso.beccari@unipg.it (T.B.)

**Keywords:** vitamin D, VDR, microglia, neurodegeneration

## Abstract

Accounting for 5–20% of the total glial cells present in the adult brain, microglia are involved in several functions: maintenance of the neural environment, response to injury and repair, immunesurveillance, cytokine secretion, regulation of phagocytosis, synaptic pruning, and sculpting postnatal neural circuits. Microglia contribute to some neurodevelopmental disorders, such as Nasu–Hakola disease (NHD), Tourette syndrome (TS), autism spectrum disorder (ASD), and schizophrenia. Moreover, microglial involvement in neurodegenerative diseases, such as Alzheimer’s (AD) and Parkinson’s (PD) diseases, has also been well established. During the last two decades, epidemiological and research studies have demonstrated the involvement of vitamin D3 (VD3) in the brain’s pathophysiology. VD3 is a fat-soluble metabolite that is required for the proper regulation of many of the body’s systems, as well as for normal human growth and development, and shows neurotrophic and neuroprotective actions and influences on neurotransmission and synaptic plasticity, playing a role in various neurological diseases. In order to better understand the exact mechanisms behind the diverse actions of VD3 in the brain, a large number of studies have been performed on isolated cells or tissues of the central nervous system (CNS). Here, we discuss the involvement of VD3 and microglia on neurodegeneration- and aging-related diseases.

## 1. Introduction

The brain undergoes a progressive increase in inflammatory state as it ages [1,2]. This altered inflammatory brain profile is linked to alterations of microglial function [3]. Similarly, microglia can undergo deterioration associated with senescence, referred to as dystrophic microglia. The morphological characteristics of dystrophic microglia differ from those of quiescent or activated microglia [4].

In the case of human microglia, they are exposed to metabolic stress over several decades due to various factors, along with continuous cycles of cell division that contribute to replicative senescence. In vitro studies have demonstrated that both human and rat microglia undergo senescence as a result of progressive telomere shortening [5,6]. Therefore, the deterioration of microglia could play a fundamental role in the development of neurodegenerative diseases associated with aging. In fact, microglia actively participate in the death of dopaminergic neurons in PD, forebrain neurons in AD, and motor neurons in amyotrophic lateral sclerosis [7,8]. The hippocampus is also vulnerable to age-related neuroinflammation [9]. Moreover, in aged brains, microglia are polarized to a pro-inflammatory phenotype (M1), with a long-lasting impairment of anti-inflammatory response (M2 phenotype) [10,11]. These observations and many others provide support for the notion that enhanced inflammatory responses during aging can be attributed, at least in part, to changes in microglial activation and function [12]. However, the degeneration of microglia in the human brain is a gradual process that unfolds slowly over time [2], marked by a spectrum of alterations with varying degrees of severity.

The active VD3, 1,25-dihydroxyvitamin D3 (1,25D3 or calcitriol), has a direct effect on gene regulation via the nuclear vitamin D receptor (VDR) [13]. Moreover, a proportion of VDR molecules are located in the cytosol [14] and have rapid modulatory effects on 1,25D3 signaling pathways, affecting phosphatases, kinases, ion channels, etc. [14]. These non-genomic actions do not require changes in gene transcription and may be ideal for modulating functional intracellular pathways activated by 1,25D3. Moreover, the VDR can act independently by VD3 modulating cellular responses [14].

In the brain, VD3 plays anti-inflammatory roles [15,16]. More generally, VD3 promotes neuroprotection through different mechanisms [16,17].

VD3 also affects the brain’s development and synaptic plasticity [18]. Maternal hypovitaminosis D induces changes in brain function and structure, causing an altered architecture of the brain at birth [19,20], and prenatal VD3 deficiency affects learning and memory processes [19,21].

VD3’s effects on neurocognition are based on suppression of inflammation and induction of neuroprotection [16,17,18]. In this context, VD3 modulates inflammation and neuroprotection by acting on the microglia.

The current review aims to summarize the role of vitamin D3 in microglia-mediated CNS brain diseases.

## 2. Microglia

### 2.1. Origin of Microglia

Microglial cell precursors develop in the yolk sac, and unlike circulating monocytes and macrophages, their development is closely related to the colony-stimulating factor-1 receptor (CSF1R) [22]. In humans, the entry of amoeboid microglial cells into the cerebral wall from ventricular space and leptomeninges begins around 4.5 weeks of gestation [23]. During early brain development, these cells migrate into the cerebral cortex in their amoeboid form. Subsequently, microglial cells transition to a ramified morphology as they migrate to the telencephalon, resembling the typical appearance of adult microglia and carrying out their functions. Circulating monocytes do not participate in the renewal of the adult microglial population. However, under specific pathological conditions, the recruitment of monocytes from the bloodstream may contribute to microglial maintenance, likely as a result of alterations in the blood–brain barrier (BBB)’s permeability [24,25].

**Figure 1:**
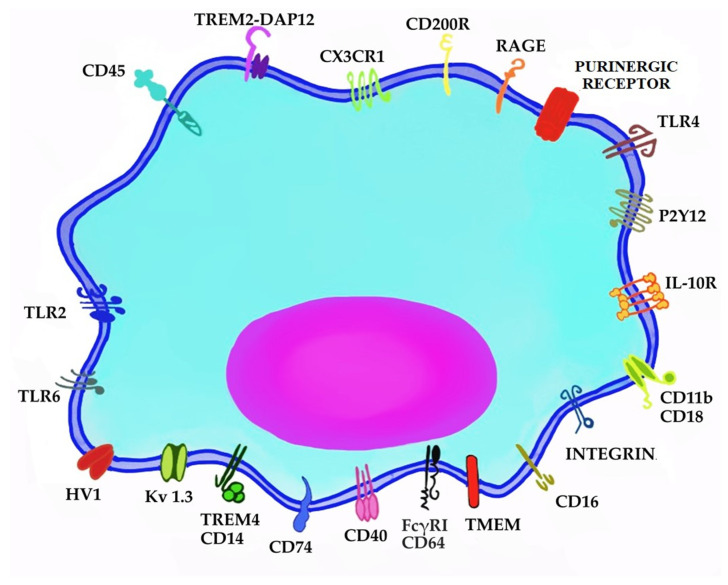
A large number of receptors regulate the activity of microglia. Stimulation of specific receptors determines different microglial phenotypes and regulates interactions with other cell types of the CNS, e.g., neurons. Moreover, the activation of these receptors is regulated in the development of the CNS, making microglia fundamental in the formation of the correct CNS architecture.

Microglia discreetly survey the brain parenchyma, maintaining homeostasis through their long and highly motile processes. When necessary, they can phagocytose pathogens, dead cells, and cell debris [26,27]. Furthermore, microglia directly communicate with neurons and oligodendrocytes through phagocytosis of cellular components and via cell–cell contacts, as well as indirect communication by releasing soluble factors [28,29]. In the adult brain, microglia play critical roles in processes such as programmed cell death, neurogenesis, synaptogenesis, synaptic pruning, and brain remodeling [30]. These functions are carried out through two main mechanisms: cytokine release, and phagocytic activity [30]. To perform such a number of functions, microglia express a large number of membrane receptors [30] (Figure 1).

### 2.2. Microglial Phenotypes

**Figure 2:**
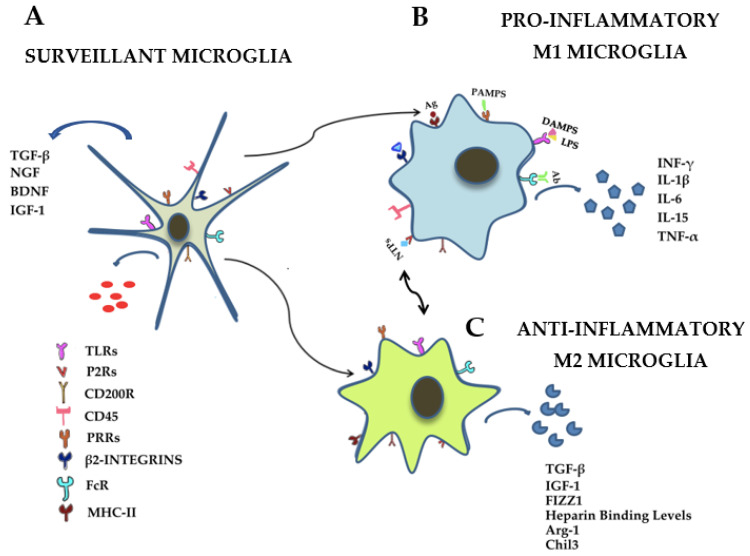
Microglial phenotypes: (**A**) In physiological conditions, microglia are surveillance cells that show a ramified morphology. The surveillance microglia are maintained by inhibitor signals, mediated by specific receptor interactions and by the release of TGF-β, NGF, BDNF, and IGF-1. (**B**) Following activation signals, microglia shift to the M1 phenotype, which is characterized by the release of pro-inflammatory cytokines and by the upregulation of the major histocompatibility complex (MHC) class II. (**C**) In order to resolve the inflammation state and limit neuronal damage, microglial cells change to the M2 phenotype, which is characterized by anti-inflammatory functions that, in turn, are characterized by the release of anti-inflammatory cytokines and other factors.

In a healthy brain, microglia exhibit a “downregulated” phenotype characterized by a branched morphology with short and delicate processes (Figure 2A). As a result, they have an increased surface area for monitoring the tissue. Each single cell scans its designated territory without overlapping or contacting neighboring microglia. Microglia interact with other brain cells, responding promptly to brain injury or damage and clearing cellular debris or accumulated metabolic products through phagocytosis [31]. Recent studies have attributed crucial roles to microglial cells in the developing brain and in adulthood, influencing synaptic pruning, synaptic connectivity, and programmed cell death, but also contributing to proper circuitry formation and brain wiring [32]. Microglia influence neuronal activity through direct or indirect interactions involving astrocytes, mediated by an adenosine-triphosphate-dependent mechanism [33,34]. Additionally, while gray-matter microglia closely associate with neuronal cell bodies to perform the aforementioned functions, other interactions may occur in the white matter, such as participation in myelin turnover [35].

In the CNS, the BBB, which is formed through intricate interactions between astrocytes’ end-feet, capillary endothelial cells (ECs), microglia, and pericytes, serves as the CNS’s largest and most stringent barrier. It effectively hinders the paracellular movement of solutes, ions, water, and proteins [36]. According to Galea and colleagues [37], the primary factors contributing to immune privilege conferred by the BBB include the unique characteristic of microglia, being the only immunocompetent elements that do not function as antigen-presenting cells. Additionally, the specialization of the adaptive immune response’s afferent arm is biased towards soluble factors rather than cell-mediated mechanisms [37]. Consequently, the movement of soluble factors (including chemokines, cytokines, lipids, small proteins, and ions) to and from the CNS plays a fundamental role in immune responses. Microglia possess nearly all of the receptors found in dendritic cells, macrophages, lymphocytes, astrocytes, and neurons [38]. Using pattern recognition receptors, (PPRs), including toll-like receptors (TLRs), microglia continuously surveil for the presence of a wide range of pathogen- and damage-associated molecular patterns (PAMPs and DAMPs, respectively) [39]. In the event of brain damage, such as disease, injury, or infection, microglial processes rapidly and independently converge on the injury sites without moving their cell body, effectively putting up a potential barrier between the injured and healthy tissue [40]. Subsequently, microglial cells undergo rapid debranching and phenotype changes, displaying distinct forms of activation known as classical (M1 phenotype) and alternative (M2 phenotype) (Figure 2A,B). In vivo, a differentiation spectrum has been proposed, with M1 and M2 representing the opposite ends [41].

#### 2.2.1. M1 Phenotype

M1 pro-inflammatory microglia (Figure 2B) play a critical role in host defense and are generally recognized as strong effector cells that are capable of eliminating tumor cells and intracellular microorganisms. Additionally, they produce substantial quantities of pro-inflammatory cytokines. However, if the pro-inflammatory phenotype is long-lasting, it can result in serious damage to healthy tissues. The pro-inflammatory cytokines that M1 microglia produce are interleukin (IL)-1β, IL-6, IL-12, tumor necrosis factor-α (TNF-α), and high levels of oxidative metabolites [42]. The neurotoxic effects can result from the continuous production of reactive oxygen species (ROS) and pro-inflammatory cytokines, as well as a dysregulated release of glutamate. However, the question regarding the neurotoxicity of activated microglia is still unresolved. According to some data [43], microglial cells play a positive role in resolving most CNS diseases. They provide evidence that, in the course of neurodegenerative diseases and aging, microglia can become harmful due to decreased expression or mutations of fundamental receptors such as CD200R, CX3C chemokine receptor 1 (CX3CR1), and triggering receptor expressed on myeloid cells (TREM) 2. This occurs when microglia deviate from their physiological behavior and become tolerogenic and hyperactive [44,45]. In aged animals, there is a decrease in microglial motility and cellular ramification processes [4]. This results in a reduced efficiency of surveillance and potentially diminished protection against tissue damage. Moreover, in the aged brain, in vitro studies indicate that microglia exhibit increased expression of MHC class II molecules and reduced sensitivity to regulatory signals, such as transforming growth factor (TGF)-β or CSF1 [46]. Throughout their lifespan, microglia can be influenced by long-lasting inflammation events and/or cytokine stimulation, leading to increased reactivity. This process of exposure to numerous harmful stimuli is known as priming. Concurrently, the accumulation of DNA mutations and damage in aged microglia can gradually result in a “hypersensitive” phenotype, characterized by heightened immune-responsiveness and reduced regulatory capacity [47].

In neurodegenerative diseases, the activation of microglial TLRs by components of dead cells can trigger the release of pro-inflammatory mediators (Figure 2B) such as cytokines and ROS. These inflammatory mediators can accelerate the death of neurons. Consequently, the inflammation induced by endogenous molecules, such as protein aggregates and dead neuron components, may contribute to the disease progression [48]. However, a large number of studies suggest that the functions of microglia are diverse. Microglia are a primary factor in establishing neuro-inflammation and neurodegeneration by releasing specific mediators for these processes [49,50]. On the other hand, microglia also contribute to CNS repair by producing neurotrophic factors and clearing myelin debris [30]. Furthermore, M1 microglia have been proposed to play a positive role in regulating neurogenesis through the production of neurotrophic mediators, such as TGF-β and insulin-like growth factor (IGF)-1 [51].

In the CNS’s neoplasm, M1 microglia’s attack is evaded by tumor cells. Studies on glioma cells have revealed that cancer cells release many factors, like IL-4, TGF-β, IL-10, and prostaglandin E2 [52], which suppress the pro-inflammatory phenotype (M1) of microglia and promote the anti-inflammatory phenotype (M2). This M1–M2 transition helps and supports cancer growth. Also, glioma cells recruit glioma-associated microglia/macrophages (GAMs) and myeloid-derived suppressor cells (MDSCs) to the tumor site and block their maturation to generate an immunosuppressed niche for tumor growth. The formation of this immunosuppressive microenvironment involves a complex interactive process among glioma cells, microglia, GAMs, and MDSCs [53].

#### 2.2.2. M2 Phenotype

M2 microglia (Figure 2C) show anti-inflammatory properties mitigating destructive immune responses and promoting wound healing [54]. The M2 phenotype is characterized by the expression of high levels of heparin-binding lectin, arginase-1 (which converts arginine to ornithine for wound healing), chitinase 3 (which prevents the degradation of extracellular matrix components), and found in inflammatory zone (FIZZ) 1, which promotes the deposition of extracellular matrix [54]. The M2 microglia play a role in prolonging neuron survival and limiting brain damage. Additionally, M2 microglia express TGF-β, IGF-1, and IL-10 [54].

An alternative approach to categorizing the M2 phenotype and function is by considering the cytokines that induce this phenotype [54]. However, it is important to note that not all cytokines that determine the M2 phenotype in macrophages have the same effects in microglia. Some M2 markers found in macrophages are not expressed in microglia. The best example is CD206, a mannose receptor [55]. Due to the distinct origins and responses of macrophages and microglia, they may play different roles in either mitigating or exacerbating diseases. Chen and Trapp (2015) [56] described microglia as crucial defenders of the CNS through development, adulthood, and illness. They highlighted that microglia’s actions are responsive and can switch from the M1 to the M2 phenotype, with the M2 phenotype playing a critical role in promoting healing [56]. Of course,, the immune responses are complex and finely coordinated in beginning and resolving every immune challenge. Unfortunately, during diseases, the immune responses can be completely impaired.

### 2.3. Microglia in Pathology

**Scheme 1:**
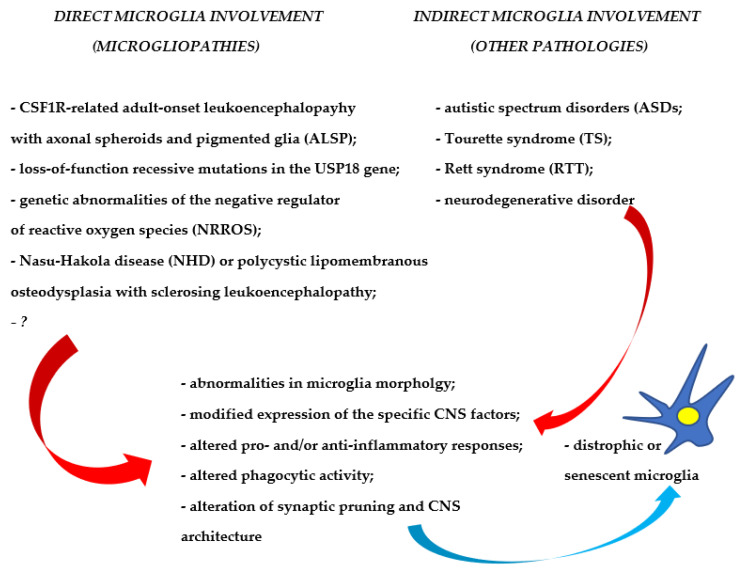
Microglial involvement in diseases: Regardless of the direct or indirect involvement of microglia in disease, the result is the alteration of microglial functions, resulting in the impairment of mechanisms fundamental to the CNS’s health. (?, other unknown pathologies).

The term “microgliopathy” has been suggested by Prinz and Priller [38], indicating microglial dysfunctions as a primary disease-causing mechanism. To date, at least four microgliopathies have been described (Scheme 1).

#### 2.3.1. Microgliopathies

CSF1R-related adult-onset leukoencephalopathy with axonal spheroids and pigmented glia (ALSP) is an uncommon type of leukoencephalopathy, classified as a form of dementia or neurodegenerative disease [57]. The clinical manifestation of ALSP is influenced by a *CSF1R* gene mutation. The CSF1R protein is a transmembrane receptor tyrosine kinase that is mainly expressed in mononuclear phagocytic cells [58,59] and functions as a homodimer. It can bind dimers of either IL-34 or CSF-1 [58,59], which modulate the function of microglia and macrophages in vivo. Additionally, this protein plays a role in regulating the proliferation, survival, and migration of these cells [60,61]. The interaction between CSF1 and CSF1R leads to complete activation of CSF1R with ERK, Akt, and Src phosphorylation [62].

Heterozygous mutation of *CSF1R* leads to a state of heightened inflammatory caused by microglial dysfunction, reminiscent of NHD (see below). Upon examination of ALSP patients’ brains, significant abnormalities are observed in the white matter, while the gray matter remains relatively intact or only mildly affected. The white matter displays demyelination and vacuolization, accompanied by the presence of axonal spheroids, peculiar astrocytes, and macrophages laden with lipids and myelin. These degenerative changes correspond to clinical symptoms experienced by patients. Typically, affected individuals display more severe white-matter abnormalities in the parietal, frontal, and temporal lobes [63,64]. 

In a study by Goldmann et al. (2015) [35] conducted on mice, it was observed that microglia express mRNAs that encode for ubiquitin-specific protease (USP18), which is abundantly and specifically expressed in microglia that are present in the white matter. Microglia located in this place are actively maintained in a quiescent state through the action of USP18. Disruption of this regulatory mechanism leads to a type I interferon (IFN)-mediated disease characterized by aggressive microglial activation and inflammation, resembling the patterns observed in other human microgliopathies [35].

Meuwissen et al. (2016) documented five patients from two unrelated families who exhibited recessive loss-of-function mutations in the *USP18* gene [65]. The *USP18* gene encodes a member of the deubiquitinating protease enzyme family that functions as both a negative regulator of type I IFN signaling and as an isopeptidase [65]. Brain ultrasound and MRI scans conducted pre- and postnatally on these patients revealed various abnormalities, including enlarged lateral ventricles, cortical malformation, cerebellar hypoplasia, malformation of the basal ganglia and posterior fossa, and more general white-matter abnormalities and calcifications. Further examination of the patients’ brains demonstrated a marked activation of the innate immune system, specifically involving the type I IFN pathway [65].

Therefore, recessive loss-of-function mutations in the *USP18* gene represent an additional cause of microgliopathy in humans.

Genetic abnormalities of the negative regulator of reactive oxygen species (*NRROS*) gene cause a serious condition that manifests in early infancy and can lead to death during early childhood [66,67]. Recently, Dong and colleagues (2020) reported on six patients from four distinct families who initially appeared normal at birth but experienced progressive disease onset within their first year of life, leading to rapid clinical deterioration. All six patients showed pathogenic *NRROS* allele variants with brain atrophy, reduced white-matter substance, and delayed myelination [65]. Smith et al. (2020) also individuated three other children in two families, with similar neuropathological features [66]. Through exome sequencing, homozygous *NRROS* variants were identified in the affected children. These observations suggest that in early postnatal brains, NRROS plays a critical role in the regulation of the development and maintenance of a homeostatic microglial subset. During development, NRROS is known to be essential for the differentiation of osteoclasts and neural cells; thus, it is also fundamental in the development of microglia [68,69]. Knockout mice lacking NRROS exhibited significant loss of microglia and axons, resulting in downregulation of the TGF-β1 pathway in microglia [70]. TGF-β1 plays a fundamental role in the CNS’s development and microglial homeostasis. These features likely form the molecular basis of the alterations described in *NRROS* KO [69,70], which parallel those observed in NRROS-deficient patients. Based on these data, Dong and colleagues proposed that NRROS downregulation may lead to uncontrolled microglial activation due to the lack of inhibitory activity of TGF-β1, with microgliosis, uncontrolled microglial activation, elevated production of ROS, chronic neuroinflammatory responses, and neuronal death [66].

NHD, also known as polycystic lipomembranous osteodysplasia with sclerosing leukoencephalopathy, was the first disorder associated with the concept of microgliopathy. NHD is characterized by severe neurological deficits and typically results in death during the fifth or sixth decade of life [71,72]. The clinical progression of NHD can be divided into four stages: latent, osseous, early neurological, and late neurological. Initially, patients exhibit subtle changes in personality that gradually worsen over months [72]. Subsequent, cognitive impairments develop, eventually leading to a late neurological state marked by profound dementia. Typically, patients succumb to respiratory or urinary infections in their fourth or fifth decade of life [71,73]. In the early 2000s, different mutations in the DNAX activation protein 12 (*DAP12*) or *TREM2* genes were discovered to be associated with NHD [72,74]. These genes encode proteins expressed in the microglia that regulate certain functions of these cells. In the absence of these proteins, microglia become excessively activated, setting off a harmful cycle of pro-inflammatory responses and neurodegeneration. Brain imaging studies of individuals with NHD have revealed severe atrophy, particularly in the frontal areas and white-matter regions [73,75]. One prominent characteristic observed in the brains of NHD patients is the widespread activation of microglia, predominantly in the frontal and temporal areas [73]. This uncontrolled microglial activation contributes to a pro-inflammatory state, impairing brain function and causing progressive brain lesions. This is believed to be the most relevant pathological component of NHD, and potentially of other microgliopathies.

#### 2.3.2. Microglia and Autism Spectrum Disorder

ASD includes many developmental disabilities defined by deficits in social interactions, communication difficulties, and repetitive behaviors. A very complex combination of genetic alteration, aberrant neurogenesis, autoantibody production, glial cell abnormalities, and epigenetic regulation is the molecular and cellular basis of this condition. Idiopathic autism, in particular, is influenced by hundreds of genetic variants. The genes associated with ASD are commonly implicated in synaptic and immune functions [76], leading to shared pathological processes, including disrupted microglial functions, synaptic alterations, and inflammation.

The presence of activated microglia, characterized by a distinct pro-inflammatory cytokine profile, was initially observed in postmortem cerebral and cerebellar cortex samples from individuals with autism, suggesting the presence of chronic inflammation in autism [77]. Another study employed an anti-Iba-1 antibody, which labels both resting and activated microglia, to analyze and quantify abnormalities in microglial cells. Microglial density was analyzed by optical techniques. This study evidenced microglial activation in 5 of 13 individuals, including two children under the age of 6. Additionally, numerous abnormalities were observed in the microglia, including soma enlargement, process retraction, and extension of filopodia [78].

The proper development of neural circuits relies on various processes, including synapse formation and their pruning. These processes are crucial for maintaining a balanced ratio between inhibitory and excitatory synapses. In individuals with ASD, this ratio is unbalanced, suggesting it as one main cause of classic ASD phenotypes [79]. An interesting research has linked impaired synaptic pruning during development to ASD [80]. However, in this study, the role of microglia was not investigated. Other studies performed on young adult ASD patients have identified the activation of microglia in specific brain areas, such as the cerebellum, orbitofrontal cortex, and anterior cingulate, which have been implicated in ASD [81]. In living ASD patients between the ages of 3 and 10, activation of microglia has also been observed in the cerebrospinal fluid, as evidenced by the increased release of pro-inflammatory chemokines [77].

A substantial body of evidence suggests a connection between inflammatory processes, subsequent damage to the neuronal circuitry, and altered neural synaptogenesis, all of which are associated with the phenotypes observed in ASD. It is possible that inherent abnormalities in microglia play a role in linking these processes, such as inflammation, disrupted neural networks, and ASD. However, further investigations are required to fully understand the relationship between the development of autism and these interconnected processes [82].

Another aspect to consider is the potential impact of maternal inflammation during pregnancy, which could contribute to the emergence of ASD-like phenotypes. Previous studies have highlighted the correlation between environmental exposure and the inherited risk for ASD [83,84]. Consequently, individuals with an inherited risk for ASD may have “primed” altered microglia that, when exposed to external inflammatory stimuli or maternal inflammation, could lead to abnormal neural network development as a result of an exaggerated microglial response [83,85].

Similarly, early stress and trauma are significant risk factors for the development of depression [86]. Exposure to stress during the early stages of life can alter immune function, not only at the time of the stressor, but also later in life. Subsequent challenges to the immune system due to stress can lead to alterations in immune, endocrine, and behavioral responses [87]. Changes in microglial functions resulting from early-life stress may play a crucial role in determining vulnerability to depression. For instance, prenatal stress, which contributes to depressive symptoms in offspring, leads to long-lasting activation of microglia in the hippocampus and increased responsiveness to systemic administration of lipopolysaccharide (LPS) [88].

TS is a neurodevelopmental tic disorder with socially inappropriate gestures or remarks. Its etiology is complex. Recent work has identified a mutation in l-histidine decarboxylase (*HDC*) as a rare but high-penetrance genetic cause of TS [89]. In mice, knockout of the *Hdc* gene, which recapitulates the molecular abnormality of disease, confirmed the validity of the model [90], showing abnormalities in microglial morphology, such as reduced arborization [91]. In *Hdc*-knockout mice, the number of microglia cells was unchanged, but there was a decrease in the number of microglia expressing IGF-1. This reduction led to impaired neurotrophic support for neurons, indicating a deficiency in microglia-mediated neuroprotection in this model. Additionally, these findings suggest an exacerbated response to environmental challenge [91]. Following LPS challenge, microglia exhibited greater activation in *Hdc*^−/−^ mice compared with wild-type controls [91]. Moreover, given the well-established role of microglia in synaptic pruning during development [92,93], the *Hdc*^−/−^ mouse model demonstrates morphological abnormalities in striatal microglia [91]. These abnormalities contribute to impaired microglial functions, leading to alterations in synaptic pruning.

In the context of Rett syndrome (RTT), microglia play a pathological role, even without immune activation. RTT is classified as a form of syndromic ASD [94]. It is primarily caused by the loss of function of the X-linked methyl CpG-binding protein 2 (*MECP2*) gene, which encodes a crucial protein involved in binding to various types of methylated DNA in the brain [95]. RTT primarily affects young girls between 8 and 16 months of age and is characterized, among other things, by slowed development. Similar to observations in autism, RTT exhibits abnormalities in dendritic and synaptic structures, suggesting a potential link between MECP2 dysfunction and autism [96].

Contrary to common belief, RTT is not solely attributed to defects in MECP2 within neurons. Studies have demonstrated that Mecp2^−/−^ microglia exhibit a significant impairment in their ability to phagocytose compared to controls [97]. Analysis of Mecp2^−/−^ brain tissue has also revealed elevated levels of cellular debris [97]. This deficiency in debris clearance may contribute to the development and progression of RTT [97].

Moreover, Maezawa and Jin (2010) demonstrated that *Mecp*2^−/−^ microglial cultures release significant amounts of glutamate into the culture medium. It is well known that high levels of glutamate can lead to impaired dendritic morphology and synaptic loss in neurons [98]. Interestingly, this phenomenon does not appear to be accompanied by an increase in pro-inflammatory cytokines [98], suggesting that microglia can induce neurotoxicity through intrinsic dysfunction. These findings align with the absence of microgliosis in RTT brains and the observed elevation of glutamate in both the brain and cerebrospinal fluid [99,100].

Even so, further studies must be performed to confirm a direct role of microglia in ASD and other behavioral patterns.

## 3. Vitamin D3 and the Vitamin D Receptor in the Central Nervous System

Calcitriol (1,25D3) is formed by sequential additions of hydroxyl groups by CYP2R1/CYP27A1 (25-hydroxylases) and CYP27B1 (1α-hydroxylase). CYP-24 negatively regulates the calcitriol level by hydroxylating and thereby inactivating calcitriol and 25(OH)D3 [101].

**Figure 3:**
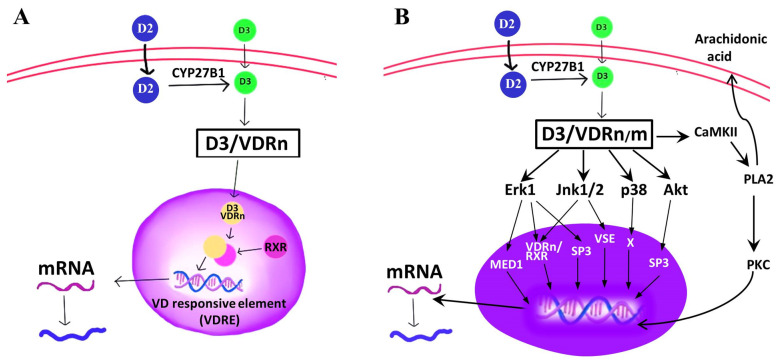
Genomic (**A**) and non-genomic (**B**) actions of calcitriol: (**A**) The classic genomic action is performed through vitamin-D-responsive elements (VDREs) located on specific promoter regions of DNA. These VDREs are activated by the VD3/VDRn/RxR complex. (**B**) Non-genomic actions, activated by modulating various cytoplasmic kinases, induce not only genetic transcription but also membrane remodeling and ion channel modulation. In this context, nuclear (VDRn) and membrane (VDRm) VD receptors are involved. VDR can also interact with various targets in the cell (see text), regulating many fundamental cellular processes.

Calcitriol binds to the nuclear vitamin D receptor (VDRn), and this complex, bound with retinoid X receptors (RXRs) as a heterodimer partner, can regulate the expression of various target genes [102,103] (Figure 3A).

In addition to its well-known role as a nuclear receptor, the VDR can also elicit rapid non-genomic effects by influencing the functions of different cytoplasmic kinases that participate in intracellular signaling pathways [14] (Figure 3B). Considerable effort has been dedicated to understanding the mechanisms underlying the non-genomic actions of VD3, including the identification of receptors involved and the potential role of VDRn. To explore these aspects, investigations have focused on the responsiveness to the calcitriol and the involvement of distinct membrane receptors. The existence of a membrane-bound VDR (VDRm) was proposed based on the observation that certain VD analogues, which are incapable of binding to VDRn, still elicit rapid responses similar to calcitriol [104,105]. Nemere et al. (2010) discovered, in the basolateral membranes of chick intestinal epithelium, a specific binding protein for calcitriol that plays a regulatory role in Ca^2+^ transport across these membranes [106].

Moreover, the VDR can exhibit non-classical and non-genomic actions though its ability to interact with various target proteins in the cell. For example, the VDR may interact with β-catenin [107], signal transducer and activator of transcription (Stat) 1 [108], c-Jun [109], Runt-related transcription factor (RunX) 1 [110], cAMP response element-binding protein [111], and the inhibitor of nuclear factor-κB (IκB) kinase (IKK) complex, which is an upstream regulator of the canonical NF-κB pathway [112]. In this way the VDR regulates many fundamental cellular processes.

The best known role of VD3 is in bone metabolism and calcium homeostasis; however, VDRs are distributed in many organs and cells, and calcitriol also regulates a wide range of biological activities in the CNS and immune system [102,113].

The enzymes involved in VD3 metabolism and biosynthesis, as well as the VDR, are expressed in both adult and embryonic brains [114,115]. In adult brains, the VDR is strongly expressed in the external granule layer in the prefrontal cortex (but also in all other layers), as well as the hippocampus (dentate gyrus, pyramidal layer of the CA1 and CA2 regions), caudate nucleus and putamen, hypothalamus, and cerebellum (granule cell layer) [115]. Very strong immunoreactivity is evidenced in the midbrain (substantia nigra, probably dopaminergic neurons) [115]. Correspondingly, high expression of CYP27B1 is found in the prefrontal cortex (internal granule layer), hippocampus, and the caudate nucleus and putamen, with less in the cerebellum; very high expression is found in the external granule layer in the prefrontal cortex, substantia nigra, and hypothalamus (supraoptic and paraventricular nuclei) [115]. However, other regions show VDR- and CYP27B1-positivity [115]. At the cellular level, the expression of these factors is evidenced in neurons and glial cells [114,115]. In rats, the expression patterns of the VDR and CYP27B1 seem similar [116].

More interestingly, microglia and neurons also synthesize VD3, which locally can operate in an autocrine and/or paracrine fashion, regulating proliferation, differentiation, and other fundamental cellular processes [114,115]. A recent work [105] showed that the VDR is mainly expressed in astrocytes, and that PDIA3—a membrane receptor of 1,25D3—is expressed in neurons and microglia. It is possible to speculate that, in astrocytes, 1,25D3 can act through the VDR, and in microglia and neurons through PDIA3, confirming the complex autocrine and paracrine interaction of this pathway.

Compelling evidence suggests that VD3 plays a role in synaptic plasticity and in brain development [18]. Animal models have demonstrated that maternal hypovitaminosis D leads to persistent alterations in brain structure and function, resulting in an abnormal brain architecture at birth, characterized by reduced cortical thickness, enlarged lateral ventricles, and modified expression of the specific CNS factors [19,20]. In support of this, previous studies have indicated that VD3 regulates the expression of numerous proteins involved in cytoskeleton maintenance, synaptic plasticity, and cellular organelles’ molecular transport [18,114,115]. Moreover, prenatal VD3 deficiency leads to enhanced long-term potentiation—a mechanism associated with synaptic plasticity, ultimately affecting memory processes and learning [19,21].

The effects of VD3 on neurocognition are mediated through multiple mechanisms, including the induction of neuroprotection, oxidative stress modulation, calcium homeostasis regulation, and suppression of inflammation. The presence of VDRs and 1-α hydroxylases is prominent in the cortex and hippocampus—crucial brain regions involved in cognitive functions such as processing, formation of new memories, and complex planning [117,118]. VD3 also influences the production of various neurotransmitters, including dopamine, serotonin, and acetylcholine [119]. In terms of dopamine, in vitro studies indicate that VD3 plays a role in the differentiation of dopaminergic neurons and influences the expression of key enzymes involved in dopamine synthesis [120]. In vivo studies have further demonstrated that VD3 deficiency alters dopamine signaling [121]. Since motor function as well as reward, motivation, and addiction behaviors are primarily dependent on dopaminergic circuits, in this context VD3 could be a fundamental factor [122].

Dopamine is produced by specific areas of the CNS, i.e., the substantia nigra and ventral tegmental area. The circuits originating in these areas are involved in motor function and the regulation of motivation and reward, respectively. Specifically, in the nigrostriatal circuit, the enhanced expression of VDRs in the striatum of mice has been associated with an increase in motivation for physical activity and other reward-driven behaviors [123,124]. This supports the notion that VD3 influences locomotor behavior, as evidenced by impaired motor function observed in mice lacking the VDR [125]. Moreover, the presence of the VDR in limbic structures, including the prefrontal cortex, amygdala, and hippocampus, suggests a potential association between VD3 and the regulation of emotional behavior and mood [126].

Finally, VD3 regulates GDNF, which safeguards against ischemia and 6-hydroxydopamine toxicity and nerve growth factor (NGF) expression, protecting against glutamate toxicity [21].

VD3 also affects the microglial function exerting anti-inflammatory effects within the brain (see Section 4).

## 4. Microglia and VD3 in Aging and Brain Disorders

During the lifetime, the physiological environment of the CNS ensures that microglia and all other cell types can carry out their functions effectively. This does not occur in neurodegenerative diseases and aging brains. In this context, microglia represent the element regulating immunological responses to external and internal stimuli. Moreover, microglia regulate the inflammatory cues in the cerebral parenchyma, being pivotal in the health of the CNS [127]. On the other hand, aging may be considered to be an increasing inflammatory state of the brain [1,2]. As mentioned above, in a growing number of studies performed on animal models, both ex vivo and in vitro, vitamin D has proven crucial for the proper function of the CNS.

So, what is the role, if any, of vitamin D in regulating the functions of microglia?

Several studies using primary microglia or microglial cell lines have shown that exogenous application of 25-hydroxyvitamin D3 (25-(OH)D3) or calcitriol affects the phenotypes of these cells, conditioning the phagocytic activities and the expression of pro-inflammatory/anti-inflammatory factors. For example, in primary microglia and BV-2 cells, 25-(OH)D3 suppressed nitric oxide (NO) production. This effect was revoked by VDR knockdown [128]. In LPS-stimulated microglial BV-2 and EOC13 cell lines, the expression of several pro-inflammatory cytokines (such as IL-6, IL-1β, and TNF-α) was also inhibited by calcitriol, as well as by inducible NO synthase (iNOS) expression [129,130].

When activated by LPS or IFN-γ, primary rat microglia produced calcitriol from 25-(OH)D3 [104]. A later study showed that CYP27A1 and CYP27B1 were expressed in rats’ hippocampus and cerebral cortex [105]. In the brain, neurons, along with microglia, are the main cell types expressing CYP27B1. Also, within the CNS, the VDR is expressed in astrocytes and, to a lesser extent, in neurons, microglia, oligodendrocytes, and ECs, even if (as mentioned above) the neurons and microglia express [105] PDIA3—a membrane receptor of 1,25D3.

Regarding the non-genomic actions, in LPS-stimulated BV-2 cells, calcitriol inhibited nuclear translocation of nuclear factor (NF)-κB and phosphorylation of extracellular signal-regulated kinase (ERK), contributing to its anti-inflammatory effect [130]. In murine primary microglia stimulated by IFN-γ, 25-(OH)D3 and calcitriol reduced the expression of IL-6, IL-12, and TGF-β while increasing the expression of the anti-inflammatory cytokine IL-10 [131]. The downregulation of pro-inflammatory factors was dependent on the suppressor of cytokine signaling (SOCS), which is induced by IL-10 [131]. In a calcitriol-treated HMO6 human microglia cell line, enhanced expression of anti-inflammatory chemokines/cytokines such as IL-10 and C-C motif chemokine ligand-17 has also been evidenced [132]. Taken together, these findings indicate that vitamin D directly influences microglial properties inducing anti-inflammatory responses. However, direct evidence for VDR involvement is limited, except in some cases; vitamin D may also exert its anti-inflammatory role through VDR-independent pathways [102,106].

Vitamin D also affects the phagocytic activity of microglia. A recent study on human primary microglia showed that the downregulation of calcitriol induced a tyrosine kinase receptor (MerTK) involved in the clearance of myelin debris and dead cells by phagocytosis [133]. On the other hand, in primary microglia obtained from vitamin-D-deficient mice with respect to control mice, the phagocytic and intracellular killing activities were significantly reduced [134].

Other in vivo results also point to an anti-inflammatory role of vitamin D. For example, in rats’ hippocampal microglia, the expression of iNOS caused by LPS injection was inhibited by local delivery of calcitriol [16], and the same results were also obtained in rats with experimental allergic encephalomyelitis [135,136]. More importantly, the alleviation of neurological symptoms was observed alongside these anti-inflammatory effects [135,137].

Neuroprotective and anti-inflammatory effects of VD3 in PD have also been demonstrated in well-studied experimental models based on the degeneration of dopaminergic neurons induced by 6-hydroxydopamine (6-OHDA)- or 1-methyl-4-phenyl-1,2,3,6-tetrahydropyridine (MPTP) in the substantia nigra [15,138]. Moreover, in the same model, vitamin D induced differentiation in HN9.10e embryonic hippocampal cells and in the hippocampus by upregulating VDR [139].

Calcitriol can affect the microglia directly or by indirect actions via other cell types, e.g., neurons. For example, the protective effect of calcitriol against ROS, glutamate, and MPTP has been demonstrated in primary cultures of midbrain dopaminergic neurons [140]. In this case, as a consequence of direct neuroprotection by calcitriol, the prevention of microglial activation in the MPTP-induced PD might be a secondary phenomenon. In this context, calcitriol induces the production of IL-34 in neurons, which, in turn, directs the microglia toward an anti-inflammatory phenotype [141].

Regarding ASD, the involvement of microglia is well established (see Section 2.3.2), but the role of VD3 in this context is controversial.

VD3 deficiency has been increasingly suggested as a risk factor for the development of ASD [119]. One of the initial randomized controlled trials that examined high-dose VD3 supplementation in children with ASD reported significant symptom improvement [142]. However, this research has raised some doubts. Conversely, another randomized controlled trial involving lower-dose VD3 supplementation in children with ASD failed to consistently demonstrate benefits [143]. Furthermore, a recent study revealed low serum levels of VD3 in 60 patients with ASD [144,145]. These discrepancies and inconsistent results observed in trials are speculated to stem from the failure to consider different forms of VD3 metabolites with varying bioavailability, as well as its receptors and binding proteins [119]. Further studies with robust methodological designs will be necessary to address and understand these discrepancies effectively.

In spontaneous hypertensive rats, chronic infusion of calcitriol into the paraventricular nucleus of the hypothalamus ameliorated hypertensive responses while also reducing microglial activation and various inflammatory parameters [146]. Another study on BV2 microglial cells demonstrated that, in the brain, the VDR regulates the brain’s renin–angiotensin system via microglial cells [147].

## 5. Conclusions

Microglia have long been considered to be a key player in the health of the CNS and, to a lesser extent, of other non-parenchymal immune cells [148]. Microglia are directly and indirectly involved in the onset of many CNS diseases (Scheme 1), and treatments modulating microglial function could be performed as new complementary therapeutic strategies in CNS diseases. However, since microglia are able to take different phenotypes—not only pro-inflammatory M1 and anti-inflammatory M2, but also a multitude of intermediate states—this goal is complex.

During aging, the loss of microglial functions is progressive, and this phenomenon causes a de facto increasing inflammatory state. If the microglial phenotypes control these processes in the brain, it is reasonable to think that the modulation of microglial activity, by limiting inflammation, could generate positive effects for brain functions. In this context, the administration of nutrients with an anti-inflammatory action in the diet makes sense.

As regards the role of calcitriol in the brain, some data are now consolidated. This role involves microglia, inter alia, and appears to be a clear anti-inflammatory role, reducing the production of inflammatory cytokines (which could have a protective effect in preventing inflammation and excessive inflammatory reactions), but also playing a role in neuroprotection. Activated microglia produce inflammatory mediator species that can damage neurons. VD3 may reduce the production of these harmful substances and promote neuronal function and survival. Furthermore, in vitro studies have suggested that VD3 promotes the differentiation of precursor cells in mature microglia, influencing their activation state. This could be important in the immune responses and outcomes of inflammation in the CNS.

While the roles of microglia in various CNS diseases are known (Scheme 1), the role of VD3 in the treatment of these pathologies is less clear. In the brain, VD3 has been involved in multiple sclerosis, sleep disorders, autism spectrum disorder, Parkinson’s disease, and Alzheimer’s disease. It should be noted that microglia are involved in all of these pathologies.

Unfortunately, treatments for these diseases with VD3 have not been conclusive, and other trials are required to fully understand its role.

The question of whether the continuous administration of VD3 in the diet can prevent the onset of CNS pathologies remains to be considered. For example, a recent study [149] demonstrated that high VD3 levels in multiple sclerosis (MS) patients reduced the risk of relapses. Since, most MS patients present low levels of VD3, VD3 supplementation is now a common practice. However, the question of whether VD3 administration prevents the onset of MS or is beneficial to patients remains unresolved. More generally, VD3 can play a complementary role in the prevention and management of neurological diseases.

Additional investigations will be necessary to evaluate the neuroprotective and therapeutic potential of VD3 in promoting healthy brain aging and preventing neurodegeneration.

## Data Availability

No new data were created for this review.

## Abbreviations

AD	Alzheimer’s disease
ALSP	Axonal spheroids and pigmented glia
ASD	Autism spectrum disorder
BBB	Blood–brain barrier
BDNF	Brain-derived neurotrophic factor
CNS	Central nervous system
CSF-1	Colony-stimulating factor
CSF1R	Colony-stimulating factor-1 receptor
CX3CR1	CX3C chemokine receptor 1
DAMP	Damage-associated molecular pattern
DAP12	DNAX activation protein 12
EC	Endothelial cell
FIZZ	Found in inflammatory zone
GAM	Glioma-associated microglia/macrophages
HDC	Histidine decarboxylase
IFN	Interferon
IGF-1	Insulin-like growth factor-1
IL	Interleukin
iNOS	Inducible NO synthase
1,25D3	1,25-Dihydroxyvitamin D3
LPS	Lipopolysaccharide
MDSC	Myeloid-derived suppressor cell
MECP2	X-linked methyl-CpG-binding protein 2
MHC	Major histocompatibility complex
MPTP	1-Methyl-4-phenyl-1,2,3,6-tetrahydropyridine
NF-κB	Nuclear factor kB
NHD	Nasu–Hakola disease
NO	Nitric oxide
NRROS	Negative regulator of reactive oxygen species
PAMP	Pathogen-associated molecular pattern
PD	Parkinson’s disease
ROS	Reactive oxygen species
RTT	Rett syndrome
RXR	Retinoid X receptor
TGF-β	Transforming growth factor-β
TLR	Toll-like receptor
TNF	Tumor necrosis factor
TREM	Triggering receptor expressed on myeloid cells
TS	Tourette syndrome
USP18	Ubiquitin-specific protease 18
VD3	Vitamin D3
VDR	Vitamin D receptor
VDRe	Vitamin-D-responsive element
